# A mixed method study exploring similarities and differences in general and social services-specific barriers to treatment-seeking among individuals with a problematic use of alcohol, cannabis, or gambling

**DOI:** 10.1186/s12913-024-11304-5

**Published:** 2024-08-22

**Authors:** Greta Schettini, Philip Lindner, Veronica Ekström, Magnus Johansson

**Affiliations:** 1 Department of Clinical Neuroscience, Centre for Psychiatry Research, Karolinska Institutet, & Stockholm Health Care Services, Region Stockholm, Stockholm, Sweden; 2 Centre for Dependency Disorders, Stockholm Health Care Services, Region Stockholm, Stockholm, Sweden; 3 Department of Social Sciences, Marie Cederschiöld University, Stockholm, Sweden

**Keywords:** Barriers to treatment, Addiction, Social services, Alcohol, Cannabis, Gambling

## Abstract

**Introduction:**

The treatment gap for addictive disorders is one of the largest in health care. Several studies have investigated barriers to treatment for different addictive disorders, but very few studies conducted have explored whether the barriers differ depending on substance or behavior or if they are common among all addictive disorders. In Sweden, addiction care is provided both by the healthcare and social services, where the latter is common, but also less popular. To our knowledge, there are no studies exploring whether the barriers are different depending on where the treatment is given.

**Aim:**

The aim was to thoroughly explore both which general and social services-specific barriers to treatment that are common, which barriers that differs, and how the barriers are described among individuals with a problematic use of alcohol, cannabis and/or gambling.

**Method:**

A mixed method convergent parallel design was conducted. For the quantitative measures, surveys including the validated Barriers to Treatment Inventory as well as questions regarding barriers in the Swedish multi-provider landscape, were collected from individuals with a problematic use of alcohol (*n* = 207), cannabis (*n* = 51), and gambling (*n* = 37). In parallel, 17 semi-structured interviews from the same population were conducted and analyzed with thematic analysis. Thereafter, the quantitative and qualitative data was compared, contrasted, and at last, interpreted.

**Results:**

The quantitative data showed that the largest general barriers in all groups were privacy concern and poor availability, and the largest barriers for seeking help from the social services was stigma, unawareness of what is offered, and fear of consequences for all groups. The qualitative data resulted in five general barriers: stigma, ambivalence, accessibility, fear of consequences, and lack of knowledge about addiction and its’ treatments, and three barriers specifically towards social services: social services reputation, fear of meeting acquaintances, and lack of knowledge. The themes were developed from data from all groups, but different aspects of the themes were mentioned by different groups.

**Conclusion:**

There are details and aspects that differentiates both the general and social service-specific barriers to treatment between individuals with a problematic use of alcohol, cannabis, and gambling, but in large they perceive similar barriers.

**Supplementary Information:**

The online version contains supplementary material available at 10.1186/s12913-024-11304-5.

## Introduction

Addictive disorders are a multi-level problem that contribute to as much as 5% of the global disease burden [19]. This not only by affecting the individual with mental and physical suffering [44], but also his or her relatives [37], as well as the society, both economically [4], and with increased criminal behaviors [5, 24, 34, 35]. It is highly prevalent: in Sweden, 4% of the adult population fulfill the criteria for an addictive disorder [1] and up to 15–20% have a harmful use of alcohol, cannabis, or gambling [36]. This reflects patterns in other countries [20–22]. With indications that help-seeking per se can increase the likelihood of recovery [14], it is concerning that only approximately 10% of individuals with addictive disorders seek and receive help for it [9, 17, 18]. This constitutes one of the largest treatment gaps among all mental disorders [31] and can be understood from various perspectives.

One perspective is structural, i.e. what is offered, and the circumstances around it, such as how long the waiting times is or how/if it is journalized. For instance, in Sweden, where addiction care is a shared responsibility between regional health care and municipal social service, it was found in a study from 2013 that only 5% of individuals with problematic drinking preferred seeking treatment from the social services as compared to e.g. psychiatric or addiction specialist treatment, which more than 50% would prefer (Andréasson et al.). The authors of the same study speculate that this could be explained by labelling addiction as a social problem, with associations to poverty or homelessness, and that this could increase stigma. The low preference for treatment at social services is particularly concerning since in many parts of Sweden, there is little region-run addiction healthcare beyond immediate detoxification.

The treatment gap for addictive disorders can also be understood by internal barriers. For the most common dependence, alcohol [19], the belief that “one should be able to make it by themselves” is a well-documented barrier [16, 46], similar to the desire for self-reliance [29, 49] reported by individuals with the most common substance use, cannabis [19]. Further, the fear of consequences is common among individuals with alcohol use disorder in the Danish population [16], and the view that seeking treatment requires total abstinence is a prevalent barrier [50]. Gambling disorder was included in DSM-5 2014, as the first, and most prevalent, diagnostic behavioral addiction [7]. One study conducted in Poland investigated if the barriers to treatment were similar for gambling disorder and other addictive disorders, showing that overall, the barriers overlapped, but it also identified specific barriers which were linked to a pervasive lack of recognition regarding the classification of gambling as a legitimate illness [12]. That unawareness and uncertainty is large and common barriers were later replicated in a study that only looked at barriers among individuals with problematic gambling [30]. Further, independent of specific addiction, stigma has consistently been reported as a major barrier [13, 47, 50].

There are large overlaps in both the psychological and neurological [39] mechanisms behind addiction of different substances, and different addictions are typically treated at the same clinics, with similar methods [15, 38, 42]. There are however also obvious differences between them, such as legal status, and the consequences the consumption can result in.

There are to our knowledge few published studies investigating common barriers across various addictions, and there are no studies focusing on the contextual perspective nor which attempts to also uncover the mechanisms behind the barriers. Therefore, research questions for the current study were:

- Are the general and social service-specific barriers to treatment and preferences on where to seek help different among individuals with a problematic use of cannabis, alcohol, and gambling (quantitative inquiry)?
- How do individuals with a problematic use of cannabis, alcohol, or gambling describe the barriers that prevents them from seeking help (qualitative inquiry)?

## Method

### Study design

**Fig. 1:**
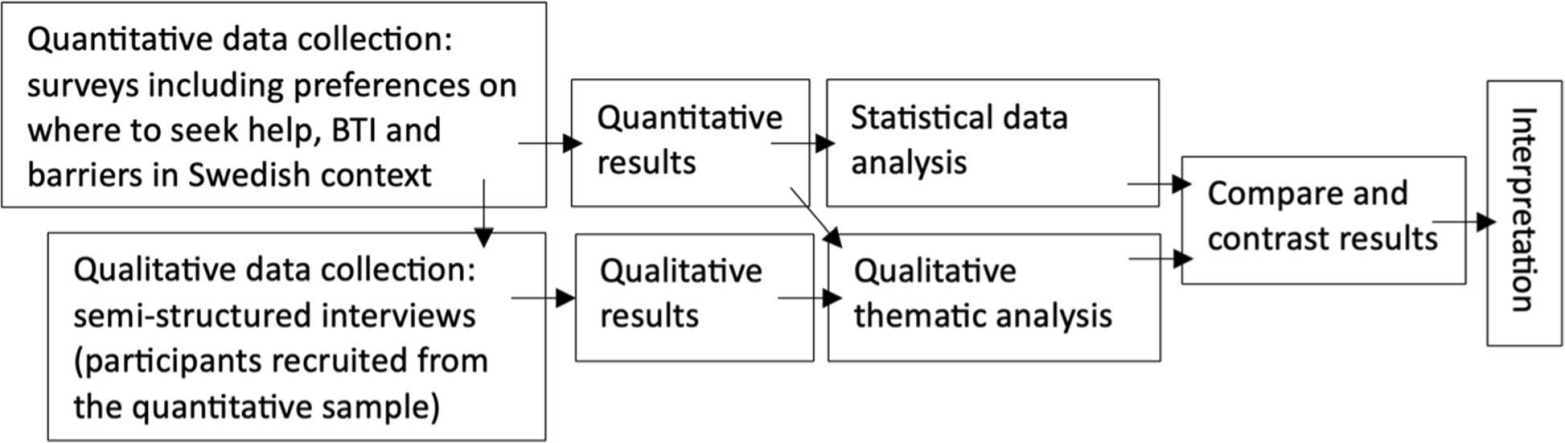
Analytic pipeline that illustrates the process from the separate data collections and analysis to interpretation

A mixed method convergent parallel design was conducted, in order to both compare the three groups (alcohol, cannabis, and gambling) statistically, and gain an in-depth understanding of any differences and similarities. This entails that qualitative and quantitative data were collected and analyzed concurrently, and then interpreted together [10, 11]. An overview of the procedure is presented in Fig. 1. The process of both designing the surveys and the interview guide was done in collaboration with the patient representative from Stockholm Center for Dependency Disorders, a region-run healthcare provider.

### Quantitative data collection and sample

The intention was to recruit participants with a problematic use of alcohol, cannabis or gambling that were considering seeking help but had not necessary sought it at time of recruitment. The main recruitment channels were therefore *alkoholhjälpen.se*, *cannabishjälpen.se*, *droghjälpen.se*, and *stödlinjen.se* (for gambling issues), which are all Swedish anonymous support-sites for addictive disorders. Recruitment was also conducted through spreading information about the study in social media. Three separate surveys with identical questions were developed, with separate links for each of the three participant groups.

For the quantitative inquiry, a cross-sectional design was used. All participants were anonymous, and the data was collected using REDCap [26, 27], a secure survey tool hosted by Karolinska Institutet, that can offer anonymity according to the GDPR definition. Links in advertisements (separate for each group, i.e. alcohol, cannabis, and gambling) directed potential participants to a landing page describing the study. Before proceeding, participants provided digital informed consent. To see all items in the survey, see Appendix 1.

The participants were asked about how many days they had consumed alcohol, cannabis, or gambling during a regular week the last year, whether they had tried to lower their consumption but failed, and if they had needed inpatient care the last year because of this consumption. All participants that consumed > 0/week, and reported problem concerning their consumption, were included. To collect the participant’s care provider preferences, they were both asked where they could see themselves seek help (where it was possible to choose multiple provider options) and their primary preference in where to seek help (only one answer).

### Barriers to treatment inventory

The surveys included the validated questionnaire Barriers to Treatment Inventory (BTI) [41], which measures self-reported barriers to seek help. BTI consists of 25 items, divided into 7 latent constructs: absence of problem, negative social support, fear of treatment, privacy concerns, time conflict, poor treatment availability, and admission difficulty. Because the constructs consist of different number of items, the mean score of each construct is used in the analyses. In this study, Cronbach’s Alpha among the different sub scales ranged from 0.633–0.829, which is considered as acceptable internal consistency reliability measures [23]. The reliability measures are presented in Table 1.

**Table 1:** Internal consistency measures for the Barriers to Treatment Inventory

Subscale	BTI 1	BTI 2	BTI 3	BTI 4	BTI 5	BTI 6	BTI 7
Cronbach’s Alpha	0.737	0.752	0.742	0.817	0.788	0.633	0.829

### Barriers specifically toward social services

Due to the previous study in a similar setting that showed the low preferences for seeking help at social service [2], the survey in the current study also included five questions regarding barriers toward social services. These barriers were based on findings on the past study, as well as clinical experience within the research group and conversations with the patient representative, and were answered on a Likert scale from 0–4, where 0 represented “do not agree at all” and 4 represented “completely agree”. One of these barriers was concerns over consequences, and if one answered > 1, representing agree to some degree, an open question popped up asking “which consequences are you worried about?” where participants could write their answers.. The answers to this open-ended question were initially planned to be analyzed as qualitative data, but the answers were short and not rich enough for this, so it was therefore instead decided to group them into categories, and quantify them according to Sandelowski et al., [45].

### Quantitative data analysis

The statistical analyses were done using R (Version 2023.09.1 + 494) [48] and Jamovi (Version 2.3.28.0) [28]. To identify differences in the constructs of BTI as well as the barriers specifically towards the social services within the groups (alcohol, cannabis, and gambling), repeated measure ANOVAs were conducted on the two forms separately. Post hoc one-way ANOVAs comparing the difference substance groups were also conducted.

### Qualitative semi-structured interviews & data collection

The semi-structured interview guide was based on previous research in a similar setting [3], conversations with the patient representative, and with minor revisions during the interview process, based on the findings that came up. The full interviews investigated several research questions, where the semi-structured questions for the present study can be found in Appendix 1.

The participants for the interviews were recruited through a nested process, where the participants that had answered the survey also could sign up to be interviewed. Initially, the aim purpose was to conduct 10 interviews per group, starting with the alcohol group and after analysis continuing with the cannabis and gambling groups. However, thematic saturation [40] was achieved (i.e. no new information between the codes emerged) after 17 interviews, where *n* = 8 had a problematic use of alcohol, *n* = 5 of cannabis, and *n* = 4 of gambling. The interviews were done over telephone between February 2023-September 2023 by first-author and clinical psychologist GS, recorded with a dictaphone, transcribed by first using Microsoft Word’s transcription feature and then re-transcribed again by GS.

### Qualitative data analysis

The subsequent process of coding, thematizing, and categorizing was done according to Braun and Clark [6]. More specifically, the transcriptions were coded by GS, with author MJ double-coding one interview and then GS and MJ having discussions about similarities and differences in the codes conducted. Initial themes were developed from the codes, with no consideration taken to participant groups. In the themes, the groups were then marked, to both observe if the themes were found in all groups and see if there were specific patterns within the groups. The codes, themes and subthemes were then discussed between authors GS, MJ, and VE. It should be noted that the process was not linear, and that the authors transitioned between coding, merging codes, thematizing and merging themes, and revising the results several times. The software used was NVivo 14.

## Results

### Quantitative results: preferences seeking help

In total, 294 participants completed the survey, and their basic demographics are presented in Table 2. A total of *n* = 198 (67%) reported that they had not yet sought help; of these, *n* = 135 (68%) reported that they could see themselves seeking help. Among the participants in this study that had not sought help, 54% could see themselves seeking help from regional health care, while only 17% could see themselves seeking municipal help, i.e. social services.

**Table 2:** Sex and age group distributions of the quantitative sample

	**Alcohol,** *n *= 207	**Cannabis,** *n* = 51	**Gambling,** *n* = 36
n women (%)	143 (69%)	2 (4%)	7 (19%)
n age category (%)			
18–29 years	16 (8%)	20 (39%)	5 (14%)
30–39 years	46 (22%)	25 (49%)	5 (14%)
40–49 years	50 (24%)	12 (24%)	11 (31%)
50–59 years	61 (29%)	2 (4%)	7 (19%)
60–69 years	24 (12%)	1 (2%)	1 (3%)
70- > years	10 (5%)	0 (0%)	1 (3%)

Preferences for first-hand preferred help did not differ between the groups. Significantly more with a problematic cannabis- and gambling use could see themselves seek help at social service specifically, compared to the participants with a problematic alcohol use; there were no other significant differences in help-seeking with specific care providers. The preferences of addiction care for those who have not sought help are presented in Table 3.

**Table 3:** Preferences regarding where to seek help for individuals that have not sought it

**Have not sought help**	**Alcohol,** *n *= 146 (72%)	**Cannabis,** *n* = 34 (67%)	**Gambling,** *n* = 18 (56%)	**Chi2-test**
Can see themselves seek help:	99 (73%)	22 (65%)	14 (78%)	*p* = **0.513**
Can see themselves seek help at… (multiple answers)				
social service	14 (7%)	10 (20%)	8 (22%)	*p* = **0.006**
other municipal care	20 (10%)	5 (10%)	6 (17%)	*p* = **0.352**
primary care	59 (29%)	16 (31%)	7 (19%)	*p* = **0.561**
psychiatry	47 (23%)	19 (37%)	14 (39%)	*p* = **0.117**
regional addiction clinic	68 (33%)	26 (51%)	16 (44%)	*p* = **0.178**
occupational care	14 (7%)	2 (4%)	4 (11%)	*p* = **0.461**
other	48 (23%)	9 (18%)	11 (31%)	*p* = **0.501**
Would at first hand seek help at… (one answer)				*p* = **0.174**
social services	9 (5%)	0	3 (10%)	
other municipal care	6 (4%)	3 (8%)	1 (3%)	
primary care	34 (21%)	8 (20%)	1 (3%)	
psychiatry	24 (15%)	5 (13%)	7 (25%)	
regional addiction clinic	42 (26%)	18 (46%)	8 (29%)	
occupational care	7 (4%)	0	1 (3%)	
other	42 (26%)	5 (13%)	7 (25%)	

### Quantitative results: barriers to treatment in general and specifically towards social services

**Fig. 2:**
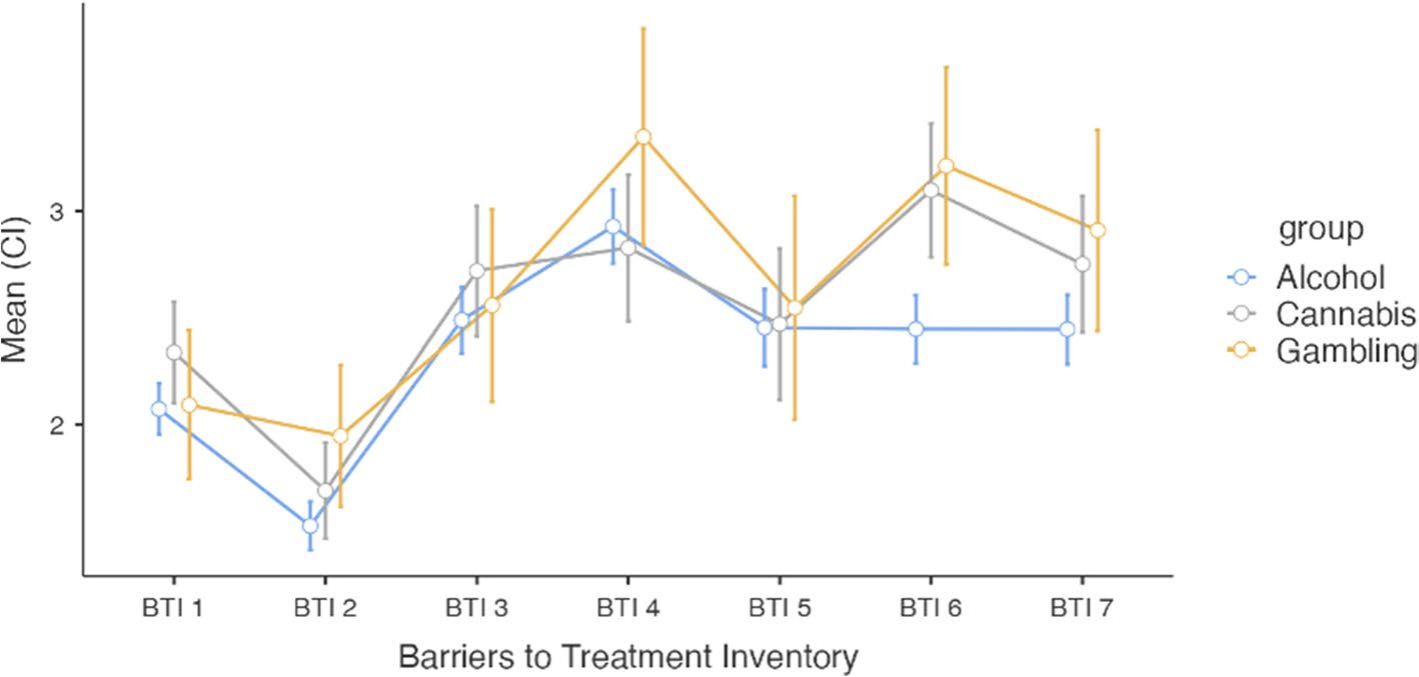
The results from the Barriers to Treatment Inventory for all three groups

The Barriers to Treatment Inventory (BTI) revealed similar patterns for all groups. As shown in Fig. 2, repeated measure ANOVAs revealed that negative social support (BTI2) was the weakest reported barrier. Privacy concerns (BTI4), such as being uncomfortable opening up about private topics with other people, along with poor availability (BTI6) were perceived to be significantly stronger barriers than the others, with one exception; that BTI4 was not significantly greater than admission difficulties (BTI7). For the full analysis, see appendix 2. Further, as shown in Table 4, there were no significant differences in any BTI scores between the groups in six of the seven subscales.

**Table 4:** Results from BTI and BTSS in all groups and the groups in relation to each other

	**Alcohol**	**Cannabis**	**Gambling**	
Barriers to Treatment Inventory (BTI): Mean & SD (Min: 0 – Max: 4)				**Between groups statistics (One-way ANOVA)**
BTI1: Absence of problem	2.07 (0.79)	2.34 (0.87)	2.07 (1.02)	F(2,48.2) = 1.9071, p = 0.160
BTI2: Neg. social support	1.52 (0.70)	1.68 (0.88)	1.96 (1.19)	F(2,43.7) = 1.8362, p = 0.172
BTI3: Fear of treatment	2.48 (1.03)	2.75 (1.08)	2.63 (1.31)	F(2,46.2) = 0.9001, p = 0.414
BTI4: Privacy concerns	2.92 (1.18)	2.88 (1.21)	3.08 (1.48)	F(2,47) = 1.0671, p = 0.352
BTI5: Time conflicts	2.44 (1.24)	2.55 (1.12)	2.66 (1.54)	F(2,47.9) = 0.0493, p = 0.952
BTI6: Poor availability	2.44 (1.04)	3.10 (1.28)	3.21 (1.27)	F(2,49.1) = 8.3281, p = < .001
BTI7: Admission difficulties	2.44 (1.03)	2.79 (1.32)	2.86 (1.34)	F(2,45) = 2.0031, p = 0.147
Barriers towards social service (BTSS): Mean & SD (Min: 0 – Max: 4)				**Between groups statistics (One-way ANOVA)**
Distrust it will work	1.44 (1.26)	1.89 (1.43)	1.89 (1.57)	F(2,45.3) = 1.6668, p = 0.200
Secrecy	0.07 (0.25)	0.19 (0.40)	0.22 (0.42)	F(2,60.6) = 3.5706, p = 0.034, posthoc A = C = G
Stigma	2.86 (1.34)	2.51 (1.50)	2.96 (1.35)	F(2,47.7) = 1.8140, p = 0.174
Unawareness	1.92 (1.53)	2.45 (1.54)	2.36 (1.54)	F(2,47.4) = 1.7940, p = 0.177
Worry about consequences	2.24 (1.71)	3.06 (1.31)	2.18 (1.72)	F(2, 258) = 5.5652, p = 0.005, posthoc C > A

**Fig. 3:**
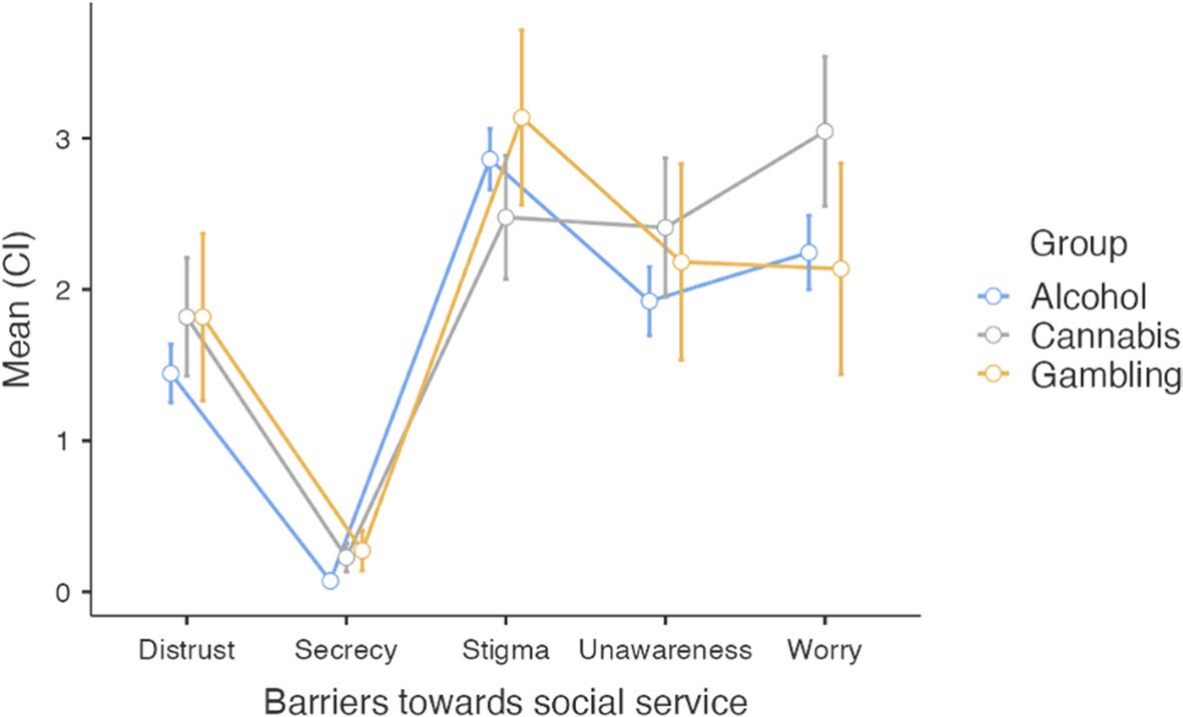
The results from the Barriers to towards social service for all three groups

Regarding Barriers toward social service (BTSS), the patterns were similar. Here, worry about the secrecy was significantly lower than all other barriers, while stigma, unawareness what was offered and worry about the consequences, were reported as large barriers. This is presented in Fig. 3, which is based on repeated measure ANOVAs, and for all analysis, see appendix 3. As visualized Fig. 3, worry of consequences was a larger barrier than stigma, the individuals with cannabis problems, which it was not for the other two groups. This is also shown significant in Table 4, where worry about consequences is reported as significantly larger for the cannabis than alcohol participants.

All those who answered positive to the question on fear of consequences were asked which consequences they were worried about. In total, 148 answers were collected and categorized. For all groups, fear of consequences regarding family, work, fear of who will find out, and fear of how one would be treated was prevalent. The fear of legal issues was not present in the gambling group, a minority in the alcohol group, while a barrier that more of half of the participants in the cannabis group reported. The results are presented in Table 5.

**Table 5:** Results from open questions about which consequences one is worried about

**Worry of consequences regarding…**	Alcohol (*n* = 88)	Cannabis (*n* = 47)	Gambling (*n* = 13)	Total (*n* = 148)
family	34% (*n* = 30)	17% (*n* = 8)	23% (*n *= 3)	28% (*n* = 41)
work	23% (*n* = 20)	32% (*n* = 15)	8% (*n* = 1)	24% (*n* = 36)
who will find out	24% (*n* = 21)	19% (*n* = 9)	31% (*n* = 4)	23% (*n* = 34)
legal issues	5% (*n* = 4)	51% (*n* = 24)	0% (*n* = 0)	19% (*n* = 28)
how one will be treated	17% (*n* = 15)	15% (*n* = 7)	15% (*n* = 2)	16% (*n* = 24)
other	11% (*n* = 10)	6% (*n* = 3)	15% (*n* = 2)	10% (*n* = 15)

### Qualitative results

When analyzing the interviews, the barriers to treatment was explored in two main categories: a) in general and b) specifically towards the social service. From these perspectives it was both explored which barriers that were overlapping for all three groups (alcohol, cannabis, and gambling), and how the groups differed from each other. The A, C, and G in the citations below refer to which group the participant belong to, and the F and M refers to the gender.

### General barriers to treatment

Five themes were identified when exploring general barriers to treatment: stigma, ambivalence, accessibility, fear of consequences, and lack of knowledge about addiction and its’ treatments. When describing why they did not seek help earlier or speculating in why others with a harmful use do not seek help, some individuals described lack of motivation, and not seeing the use as harmful. However, since none of the participants described experiencing this as a present barrier, this was not included as a theme.

#### Stigma

Stigma is a well-known barrier to treatment [13, 47, 50], which was confirmed in these interviews for all the groups. These interviews did however reveal that what one was ashamed of depended on which group one belong to. For those with a problematic alcohol use, the stigma was related to feeling weak and having a bad character and appear to be “someone that cannot handle life on their own” (A13F), where the stigma in the group of cannabis users instead was related to that buying and using is a criminal act, and the social view on this, for instance described like “if someone is an alcoholic, you feel sorry for them, but if someone is a junkie, they are felonious” (C159M). For those with gambling issues the stigma is rather directed to one’s relatives “all with gambling addiction that I have talked to say that their biggest fear is the guilt, knowing that one has done something wrong and expose one’s loved ones to stuff you do not want to expose them to…” (G10M).

#### Ambivalence

This theme reflects the ambivalence participants in all groups described on giving up the function that the substance serves. In all groups the substance filled the function of escaping anxiety and loneliness, described such as “I am not feeling good, I never have, but when I am high, time disappears” (C159M), “I gamble because I am lonely” (G17M), and “alcohol is the best way to suppress anxiety” (A11F), and quitting using means one must deal with this in another way. It was also described how the substances fills other, different functions depending on group. Here, alcohol was described as filling a social function, cannabis was used for both creativity and pain relief, and continuing gambling gave the hope of winning back all money that one has lost. Another factor in this theme is that seeking help will imply setting goals, and the ambivalence on committing to this with the risk of failing. One participant described that “I feel that I can’t bear to disappoint myself … and when one is trying to get clean that is what happens, because there will be relapses … and for every relapse, it hurts even more” (C156F).

#### Accessibility

Accessibility was a barrier described in all groups. A few participants mentioned that when one seeks help, the waiting list is too long, and one described that “to have to describe the situation and tell the story for like three different people before one receives the right help” (G19M) was a barrier. One alcohol participant said that “When one finally found something… well, then the opening hours are only a few days a week … and many that drinks do it every day but especially during the weekends, and then you can’t find almost any place to reach out for help” (A18F), and also in the gambling group it was also described how one often sits and gambles in the middle of the night and the help offered is available between a small range of time during the day when one is at work. Here, both groups describe a barrier that the help is not offered during the time where it is the most risk that they will relapse. A pattern that was only described in the gambling group was that “there are small gaps where one feel like “I need help!” and if help would be as visible as the gambling advertisement with banners one would click at it right away … but when help lines are closed or there is a long waiting line you just shut your computer or hang on the phone and maybe try again in six months” (G19M).

#### Fear of consequences

Seeking help will include admitting that one has problems, and the fear that this will not only imply receiving help, but also other, negative consequences was mentioned as a large barrier for all groups. Among the alcohol and cannabis groups, the fear of losing one’s driver’s license was such a consequence, as well as among parents, the fear of receiving a report of concern regarding one’s child(ren). In turn, this comes with the fear that “one can actually loose custody of the children if it is really bad” (A52M) or that “what if the social service would come knock at the door? When you live in a smaller village and there is an unknown car coming up with two ladies, the neighbors will start wondering and the rumors will begin spreading” (A2F).Further mentioned consequences were not receiving other psychiatric care when the caregivers find out about the use, and that one’s boss will find out about the use and that it will affect one’s working situation. However, neither of these consequences were mentioned in the group of gamblers. They did however describe the fear of the consequences when relatives find out: “especially if one has a family, then you are so afraid they will leave you, which you absolutely think that they will” (G10M). Among those with a problematic use of alcohol and cannabis it was also a fear for the consequences when relatives find out, but more that they will look at you in a different way, with examples from interviews like “my children will not need to feel that they have a mom that is an alcoholic” (A6F) and “if my wife’s parents would find out that I use cannabis… I mean, they already hate me so…” (C159M).

#### Lack of knowledge about addiction and its treatments

In the gambling group, one mentioned barrier is the unawareness that gambling addiction is stated as a real psychiatric problem that one can receive help for. This is not mentioned in the other groups, but instead the unawareness that it is possible to receive help to lowering, but not necessary 100% quit, one’s consumption. One participant from the cannabis group stated that: “there is a fear when I seek help, that it implies that I should be sober for the rest of my life… and also include everything (like taking a beer)” (C150M), and a participant from the alcohol group said that “I am not ready to fully quit, so I am trying to lowering the consumption on my own” (A55F).

### The barriers to treatment specifically from the social service

Three themes were identified when exploring barriers to treatment specifically from social services. These themes were social services reputation, fear of meeting acquaintances, and lack of knowledge.

#### Social services reputation

When asked about barriers to seek help from social service specifically, it was described that if there is a stigma in general seeking help for addiction, it is even more stigmatizing seeking it from the social services: “it feels like the social service is somehow related to extreme misery” (C150M) and “the social services, is related to… well maybe that you feel like a loser if you end up there” (A52M). Participants from the alcohol and cannabis groups also stated that they had heard or read about negative narratives related to social services and having close one’s with negative experiences. This theme was not as clearly appearing in the gambling group, since the social services can help with debt settlements and financial aid, which some of the gambling participants have received.

#### Fear of meeting acquaintances

For all groups, one barrier to seek help from the social service was that these are more local, and in smaller villages it is a worry to meet someone that one knows: “I know every social worker in this municipality, and I will not go to them with my problems, I just won’t… and that barrier needs to go away in order for the addiction care to work, because there will always be a risk in smaller municipalities that you will run in to your therapist” (C93M). Another participant described that “the rumors that “she is an alcoholic” can become a tragedy for a family or an individual” (A55F).

#### Lack of knowledge about social services and the prerequisites

When asking the participants of barriers to seek help for their use at social services, many answered with both surprise that help was offered from the social service; “Why do they not make themselves heard that there is help there to get?” (G87M) and with skepticism on what this help implied “seeking at the social service? Never! You never know what they are up to… will they force you to compulsory care? Will they steal my driver’s license? What happens?” (A13F). This was clearly described in relation to cannabis: “when using a substance that is also illegal, one really needs to get information on what can happen (in order to seek help)” (C150M).

## Discussion

The present study revealed that individuals with a problematic use of alcohol, cannabis, and gambling perceived similar barriers to seeking treatment, both in general and with social services specifically. When asked to describe these barriers, themes were superficially similar; however, key between-group differences also emerged in terms of group-dependent subthemes.

### Similarities between groups

We were not able to find any significant differences between groups regarding preferences on where they would firsthand seek help, although it should be noted that some of the pairwise contrasts were not powered to detect small differences. In all groups, (regional) health care was more popular than (municipal) social services. One prominent theme that emerged from the qualitative interviews was the reputation of social services, with individuals from all – and especially in the alcohol and cannabis groups – describing it as more stigmatizing seeking help from social services. Another theme was the lack of knowledge about social services, both regarding care offered as well as the prerequisites for receiving treatment. Further, in the quantitative questions regarding barriers toward social services (BTSS), fear of consequences was reported as a large barrier among all groups. In the open question about which consequences one feared if seeking help at social service, fears regarding family members, colleagues, and similar finding out, and worry about how one would be treated, were frequent in all three groups.

Both the qualitative themes, and the complementary open questions, help explain the quantitative preference ratings. Notably, the earlier study by Andréasson, et al. (2013), that also investigated preferences, found that as few as 5% of their participants (individuals in Sweden with a problematic alcohol use) preferred seeking help from the social services. The present study replicates these results and extends them by including individuals with a problematic use of gambling or cannabis. The present study also offers deeper insight into why so few prefer seeking treatment from social services, with both the interviews and the open-ended answers from the surveys indicating that the fear of meeting acquaintances as well as the worry of receiving a report of concern, or loosing custody of one’s children, is what keeps people from seeking help from social service. In Sweden, social services have a broad set of responsibilities that include exercising public authority such as rehoming children, which (regional) health care cannot. The present study’s results indicate that this dual role explains why so few would choose to seek addiction help from social services.

Of note, there were one significant difference between groups in general barriers to treatment-seeking (BTI), where poor availability being top-ranked barriers among the gambling and cannabis groups, but not the alcohol group. This could reflect that there actually are more help available for the alcohol group. Regarding rated barriers to treatment seeking at social services specifically (BTSS), no significant differences were found in the questions concerning distrust that the treatment would work, stigma, or unawareness, the latter two being rated high. These results are not only congruent with uncovered qualitative themes around stigma and accessibility, but also the themes around lack of knowledge about addiction and its treatments, as well as lack of knowledge about social services and the prerequisites. Our findings support the need for future initiatives to lower stigma, make treatment more available, and as well as reaching out with information about both addiction and its treatments, to attempt to lower the well-known addiction treatment gap.

### Differences between groups

In the present study, we also uncovered important differences between the groups. The alcohol group rated themselves significantly less likely to seek help at social service compared to the other groups. Further, the cannabis group was the only one that rated fear of consequences to be a larger barrier than stigma. In the open question about which consequences that one feared if seeking help at social services, the fear of legal consequences was the most reported factor in the cannabis group; this fear was only mentioned a few times in the alcohol group and not mentioned at all in the gambling group. In the qualitative data, the fact that cannabis use is illegal in Sweden was mentioned both in conjunction with the theme of stigma, in the fear of consequences, and in lack of knowledge about social services and the prerequisites. This would appear to suggest that decriminalization cannabis could lower barriers to seek treatment, but according to a recent systematic review, there seem to be no indications of such trends in jurisdictions where cannabis has been legalized in the last decade [8].

There were also differences between the groups in the themes uncovered in the qualitative data. Other than the ambivalence on losing an escape from anxiety or loneliness participants, all groups also described ambivalence on giving up other perceived functions of their consumption, but the different groups described different types of functions. Similarly, participants from all groups talked about stigma as a major barrier, but in different ways. In many of the themes, the alcohol and cannabis groups were similar, while the gambling group differed more. For instance, regarding accessibility, only gamblers described short, intense, and transient moments when motivation to seeking help was high – if help is not easily available then, motivation to seeking help would be lost. They also differed in describing fear of consequences, which were more oriented towards how it would affect their relationships with close relatives rather than the fear of being labeled as an “addict” in their social community. In difference to the other groups, they did not either mention the fear that their gambling would result in an exercise of public authority.

This pattern, with the gambling group deviating from the others, likely stems from it being a behavioral addiction instead of the use of a substance. Practically, a behavioral addiction does not automatically result in e.g. being unsuitable for driving vehicles or taking care of children. This could explain that worry about receiving a report of concern or losing custody regarding one’s child(ren) was not described as a fear of consequence in the interviews among gamblers, to the degree that it was in the other groups. Further, even though all groups described worry about their relatives being disappointed, the gambling group was the only one expressing worry about their relatives also leaving them when finding out about the use. This could be explained by the fact that the cessation process differs between gambling and other addictions: someone quitting gambling has typically not only lost (large amounts of) money, but also have large debts that requires numerous years to pay back [33], in turn affecting the everyday life for both oneself and most probably also one’s relatives. The gamblers were also the only ones describing their windows of motivation to seek help to be short, intense, and then closed if help is not available right away. One difference between gambling disorder and other addictions is that quitting predicts higher suicidality [32], which is in turn related to giving up the hope of winning back money lost. This severe struggle to quit is unique to gambling addiction, which in turn could be one possible explanation for the short, and intense motivation gaps closing so quick.

In the study conducted in Poland [12], that also compared barriers to treatment-seeking among gamblers to other addictions, it was found that the largest difference from the other addictions was the lack of knowledge that gambling addiction is defined as a psychiatric diagnosis, and that there was a lack of adequate help offered. The results from the present study can be seen as a replication of this, although these findings were not as prominent. One possible explanation for this could be that gambling addiction has seen a better integration into the health care landscape in the last few years, at least in Sweden [25]. However, gambling is the most recently added addiction in the Diagnostic and Statistical Manual of Mental Disorders [43], which could explain it being perceived as difficult to find help for, since fewer professionals are focused on it. This might also contribute to the fear of relatives finding out, with it still being less established as a psychiatric problem in society.

### Future research and limitations

Considering the quantitative and qualitative results jointly, one driver behind many of the barriers appears to be a lack of information and lack of transparency on how treatment provision works. This not only applies to the quantitative results such as unawareness ratings, but also to the privacy concerns, as well as barriers such as fear of treatment and fear of consequences. Exploring the effects of transparency initiatives appears to be promising avenue for future research. Also, because there were few differences in perceived barriers to treatment between the groups, this could imply that successful initiatives to lower the treatment gap in one group ( e.g. alcohol users) could be tested, with small alterations, also among other groups. Further, this study focused on barriers between groups with different primary use. It encouraged to continue exploring both barriers to treatment being moderated by other factors than which problematic use one has, such as gender, age, or other social factors. Also, our open questions regarding barriers revealed themes that the validated BTI instrument [41] does not cover, such as the fear of consequences or self-stigma. Findings from the current study indicate the need for a new or revised version of this and similar instruments, to cover a broader spectrum of perceived barriers.

A strength of the study is the amount of both quantitative and qualitative data, giving a strong foundation for interpretation. There are also several limitations of the current study, one being the between-group difference in sample sizes. Since statistical power of a given contrast is primarily driven by the size of the smaller group, we cannot rule out that more differences would have been revealed with larger groups, in particular problem gamblers. The groups were however recruited from similar contexts and were overall similar, and population-level difference in prevalence rates of the different addiction groups entail that differing sample sizes are not unexpected. To minimize the demography items, to increase response rates as well as for anonymity reasons, no information about co-morbidity was collected. This makes it possible in theory for participants with a problematic use of more than one of the substances to answer the survey twice or even three times, visiting it from different sites, which would render reported values statistically dependent. However, open-ended questions did not in any way signal this to be the case, nor were participants reimbursed for their participation, entailing no incentive to participate multiple times.

Other limitations include that we only investigated barriers in general, and specifically towards social service, but not barriers specific to other care providers such as the health care. Including this would have increased insights into broader, provider-specific barriers. Lastly, the included participants were recruited from help-seeking sites. Even though it could be considered a strength to include individuals that wanted help but who had not yet sought it, the sample still only represent a minority of the broader group that we aimed to reach.

## Conclusion

This study uncovered similarities and differences in perceived barriers to treatment-seeking among individuals with a problematic use of alcohol, cannabis, or gambling, and offers some in-depth insight into what may be needed to overcome them. There are details and aspects that differentiates both the general and social service-specific barriers to treatment between individuals with a problematic use of alcohol, cannabis, and gambling, but in large they perceive similar barriers. This suggests that initiatives proven effective in reducing treatment barriers to in one addiction group may be used as guidance for other groups, albeit with some tailoring.

### Supplementary Information

Supplementary Material 1

Supplementary Material 2

Supplementary Material 3

## Data Availability

Data will be made available upon reasonable request to the corresponding author.
