# Supplementary material for: A mixed method study exploring similarities and differences in general and social services-specific barriers to treatment-seeking among individuals with a problematic use of alcohol, cannabis, or gambling
Source: BMC Health Serv Res. 2024 Aug 22;24:970. doi: 10.1186/s12913-024-11304-5 (PMC11342637; doi:10.1186/s12913-024-11304-5)
Supplement: Supplementary file 1 — Supplementary Material 1 [file 12913_2024_11304_MOESM1_ESM.pdf]

SURVEY

- Gender?
- Age?
- How many days do you [consume alcohol/consume cannabis/gamble with money] a regular week (the last year)?
- Have you tried to reduce your [alcohol consumption/cannabis consumption/gambling with money] the last year but not succeeded?
- Have you needed emergency care and/or been hospitalized the last year due to your [alcohol consumption/cannabis consumption/gambling with money]?

- Have you sought help for your [alcohol consumption/cannabis consumption/gambling with money]?
  - If yes: where have you sought help for your [alcohol consumption/cannabis consumption/gambling with money]?
- Where could you see yourself seek help for your [alcohol consumption/cannabis consumption/gambling with money] (multiple answers possible)?
- Where would you at firsthand seek help for your [alcohol consumption/cannabis consumption/gambling with money]?

BARRIERS TO TREATMENT INVENTORY

This was answered on a likert scale from 0-4, where 0 represents "do not agree at all" and 4 represents "completely agree".

Read the statements below and fill in how well you agree. Here, treatment refers to all help given by professionals.

(Absence of Problem)

- I do not think I have a problem with [alcohol/cannabis/gambling]
- No one has told me I have a problem with d[alcohol/cannabis/gambling]
- My [alcohol usa/cannabis use/gambling] is not causing any problems
- I do not think treatment will make my life better
- I can handle my [alcohol usa/cannabis use/gambling] on my own
- I do not think I need treatment

(Negative Social Support)

- I will lose my friends if I go to treatment
- Friends tell me not to go to treatment
- People will think badly of me if I go to treatment
- Someone in my family does not want me to go to treatment
- My family will be embarrassed or ashamed if I go to treatment

(Fear of Treatment)
- I have had a bad experience with treatment
- I am afraid what might happen in treatment
- I am afraid of the people I might see in treatment
- I am too embarrassed or ashamed to go to treatment

(Privacy Concerns)
- I do not like to talk in groups
- I hate being asked personal questions
- I do not like to talk about my personal life with other people

(Time Conflict)
- I have things to do at home that make it hard for me to get to treatment
- It will be hard for me to find a treatment program that fits my schedule

(Poor Treatment Availability)
- I am moving too far away to get treatment
- I do not know where to go for treatment
- I have difficulty getting to and from treatment

(Admission Difficulty)
- I will have to be on a waiting list for treatment
- I have to go through too many steps to get into treatment

BARRIERS TO TREATMENT SOCIAL SERVICES

This was answered on a likert scale from 0-4, where 0 represents “not at all” and 4 represents “completely”.

How much do the aspects below prevent you from seekeing help for your [alcohol consumption/cannabis consumption/gambling with money] at the social services or in other ways by the municipal:

- I do not think the interventions offered will help me
- I feel insecure about secrecy
- I feel ashamed
- I do not where to turn to seek help
- I am worried about the consequences of social services finding out about my [alcohol consumption/cannabis consumption/gambling with money]
    - If <2: Which consequences are you worried about? (open answer)

The survey also included two other open questions, not used in the present study.

INTERVIEW GUIDE

The qualitative interviews investigated several research questions, where the semi-structured questions for this study was:

- What barriers do you think there are to seek help for one’s problematic [alcohol consumption/cannabis consumption/gambling with money] in general?
- What barriers do you think there are to seek help for one’s problematic [alcohol consumption/cannabis consumption/gambling with money] at the social services?
