# Supplementary figures and images for: A mixed method study exploring similarities and differences in general and social services-specific barriers to treatment-seeking among individuals with a problematic use of alcohol, cannabis, or gambling
Source: BMC Health Serv Res. 2024 Aug 22;24:970. doi: 10.1186/s12913-024-11304-5 (PMC11342637; doi:10.1186/s12913-024-11304-5)

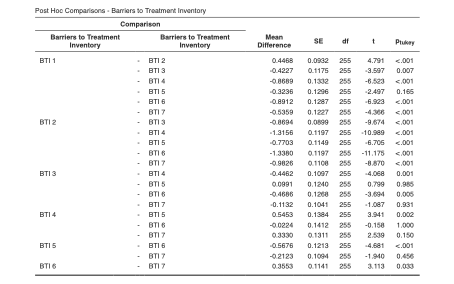

Supplement: Supplementary file 2 — Supplementary Material 2 [file 12913_2024_11304_MOESM2_ESM.png]

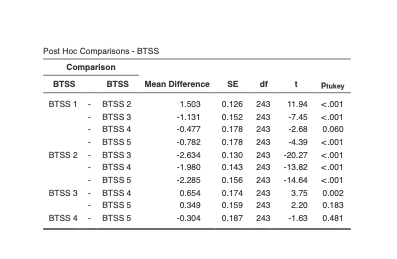

Supplement: Supplementary file 3 — Supplementary Material 3 [file 12913_2024_11304_MOESM3_ESM.png]
